# Exploring the mechanisms of weight loss in the SHED-IT intervention for overweight men: a mediation analysis

**DOI:** 10.1186/1479-5868-6-76

**Published:** 2009-11-18

**Authors:** David R Lubans, Philip J Morgan, Clare E Collins, Janet M Warren, Robin Callister

**Affiliations:** 1 School of Education, Faculty of Education & Arts, University of Newcastle, Australia; 2 School of Health Sciences, Faculty of Health, University of Newcastle, Australia; 3 Danone Baby Nutrition, Trowbridge, UK; 4 School of Biomedical Sciences, Faculty of Health, University of Newcastle, Australia

## Abstract

**Background:**

Statistical mediation analysis can be used to improve the design of obesity prevention and treatment programs by identifying the possible mechanisms through which an intervention achieved its effects. The aim of this study was to identify mediators of weight loss in an Internet-based weight-loss program specifically designed for overweight men.

**Methods:**

The Self-Help, Exercise and Diet using Information Technology (SHED-IT) program was a 3-month randomized controlled trial (Internet-based intervention group vs information only control group) that was implemented in 2007 with baseline and 6-month follow-up assessment of weight, physical activity and dietary behaviors. Intention-to-treat and per-protocol mediation analyses were conducted using a product-of-coefficients test.

**Results:**

Participants (N = 65) were overweight and obese male academic (*n *= 10) and non-academic (*n *= 27) staff and students (*n *= 28) from the University of Newcastle, Australia. Mean (SD) age = 35.9 (11.1) years and mean (SD) BMI = 30.6 (2.8). In the intention-to-treat analysis, both groups lost weight, but relative to the control group, the intervention did not have a statistically significant 'total effect' on weight, τ = -.507, *p *= .716 (95% CI = -3.277 to 2.263). In the per-protocol analysis, the intervention had a statistically significant 'total effect' on weight, τ = -4.487, *p *< .05 (95% CI = -8.208 to -.765). The intervention did not have a statistically significant effect on any of the hypothesized mediators and none of the behavioral variables mediated weight loss in the SHED-IT program. Although participants in the intervention group reduced their fat intake over the study period, the changes did not satisfy the criteria for mediation.

**Conclusion:**

Few studies have examined the mediators of weight loss in obesity treatment interventions. While none of the hypothesized mediators satisfied the criteria for mediation in the current study, there was some evidence to suggest that overweight men in the SHED-IT intervention reduced their fat intake over the study period. Future obesity treatment and prevention programs should explore behavioral mediators of weight loss using appropriate statistical methods.

**Trial Registration:**

Australian New Zealand Clinical Trials Registry No: ANZCTRN12607000481471.

## Background

Obesity is a major cause of preventable death and is associated with substantial direct and indirect health care costs [1]. While the relative contribution of physical inactivity and poor nutrition to obesity prevalence is controversial [2], it is irrefutable that the obesity pandemic is a product of widespread energy imbalance [3]. The treatment of obesity is problematic and weight loss interventions generally result in modest effects [4]. The lack of success in interventions may be due to limitations in study design and intervention components, program implementation, effect moderation and measurement issues [5].

**Figure 1:**
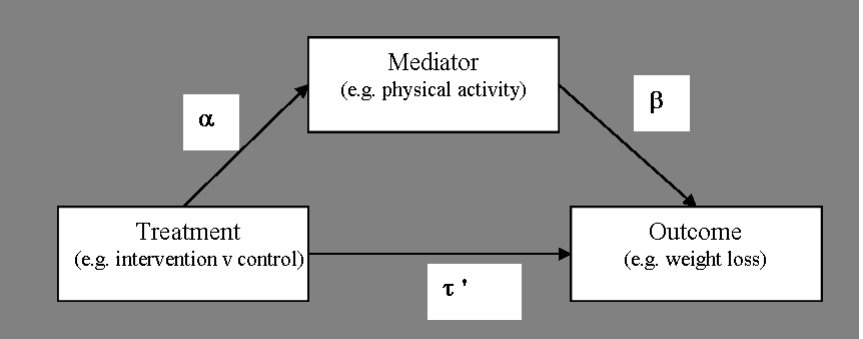
**Overview of mediation analysis**.

Statistical mediation analysis can be used to improve the design of future interventions by identifying the possible mechanisms through which an existing intervention achieved its effects [6,7]. Mediation analyses provide information regarding the effectiveness of various intervention components and such information can be used to tailor interventions for specific groups. Furthermore, mediation analyses allow researchers to develop more parsimonious interventions by eliminating less important components and emphasizing others. In its simplest form (Figure 1), mediation analysis involves adding a hypothesized mediating variable (e.g. physical activity) to the regression equation including one independent (e.g. intervention condition) and one dependent variable (e.g. weight loss) [8]. While intervention studies often report their effect on potential mediators, few studies have used mediation analysis to determine if changes in the hypothesized mediators were responsible for changes in the primary outcome. For example, previous weight loss interventions have evaluated their impact on the physical activity levels of their participants [9,10]. Other studies have used change score correlations to examine the relationship between changes in weight and changes in physical activity [11,12]. However, neither of these two approaches conclusively establishes whether weight loss could be attributed to increases in physical activity.

In mediation hypotheses, it is assumed that the inclusion of a mediating variable (e.g. physical activity) will reduce the magnitude of the relationship between the independent (i.e. treatment condition) and dependent variables (e.g. weight loss) [13]. Mediation analyses have been used in a number of studies to evaluate the theoretical framework of physical activity and dietary interventions, but most have only assessed psychosocial constructs (e.g. self-efficacy) [7,14,15]. Similarly, mediation analyses in weight loss interventions have generally focused on psychosocial mediators [16,17], which helps to determine the construct validity of an intervention. Mediation analyses have been recommended to improve the design of obesity prevention trials [18,19] and we suggest that obesity treatment interventions may similarly benefit from mediation analyses.

The primary aim of this study was to identify behavioral mediators of weight loss in an Internet-based intervention for overweight men. The Self-Help, Exercise and Diet using Information Technology (SHED-IT) program was an Internet-based weight loss program designed specifically for men. The trial was registered: Australian New Zealand Clinical Trials Registry No: ANZCTRN12607000481471.

## Methods

### Study design and sample size

SHED-IT was an assessor-blinded randomized controlled trial (RCT) that adhered to the CONSORT protocol [20]. Overweight or obese (BMI between 25 and 37 kg/m^2^) male staff (academic and non-academic) and students aged 18 to 60 years were recruited from the University of Newcastle from advertisements placed on University notice boards and the University website in late August 2007. Participants were blind to group allocation at baseline assessment and assessors were blinded to treatment allocation at all time points. Based on 80% power to detect a 3.0-kg difference among groups as significant (*P *< .05, two-sided), a sample size of 18 participants for each group was needed. Assuming a 20% attrition rate, a total sample of 45 subjects was required. Participants were randomly allocated to one of two groups: an Internet-based weight-loss group or an information only control group using a computer-based random number-producing algorithm.

### Intervention details

The SHED-IT (Internet) intervention involved one face-to-face session followed by three months of online support. Participants attended an introductory session (75 minutes) led by one of the male researchers in September 2007. A detailed description of the study design, intervention components and major findings have been published elsewhere [21]. The first 60 minutes of the information session focused on the modification of diet and physical activity habits and behavior change strategies including self-monitoring, goal setting and social support, based on Bandura's Social Cognitive Theory [22]. The second part of the session (15 minutes) focused on how to use the free study website http://www.calorieking.com.au.

The control group attended one information session which was identical to the intervention group, but without the 15 minute description of the online component. The same male researcher delivered the information session. Separate sessions were conducted for intervention and control participants to avoid contamination between study arms. All participants were provided with an information booklet focusing on key behavioral strategies to achieve a healthy weight. The key behavioral strategies were as follows: (i) learn to read food labels, (ii) keep a healthy lifestyle diary, (iii) plan your meals, (iv) reduce your junk food snacks, (v) reduce your portion sizes, (vi) beware of beverages, (vii) surf the (hunger) urge, (viii) every step counts and (ix) tilt the balance with physical activity.

### Outcomes

Outcome measures were obtained from participants at baseline (September, 2007), 3-months (December, 2007) and 6-months (March, 2008) after the start of treatment. Baseline and 6-month data are presented in this paper. The 3- and 6-month study results are reported elsewhere [21]. Measurements were taken at the University of Newcastle (New South Wales, Australia) using the same instruments and the same trained assessors at each time point. Baseline assessments were taken 1-2 weeks before the information session. Details of age, occupation and socioeconomic status (SES) were also collected. SES was based on postal code of residence [23]. The primary outcome was change in body weight (kg and percent change from baseline). Weight was measured in light clothing without shoes to 0.1 kg, using CH-150 kp digital scales (A + D Mercury Pty Ltd, Australia). Secondary outcome measures of diet and physical activity were assessed. Dietary behavior was measured using the Dietary Questionnaire for Epidemiological Studies (DQES) Version 2 Food Frequency Questionnaire (FFQ) from the Cancer Council of Victoria [24]. The FFQ provides an estimate of daily energy, macronutrient intake, average meal portion size, total kilojoule intake and alcohol consumption. FFQ scores were computed using NUTTAB 1995, a food composition database of Australian foods (Australian Government Publishing Service, Canberra) with software developed by the Cancer Council of Victoria. The development of the DQES (13) and its use in a cohort of over 40000 adults (>17000 men) have been reported previously (14). The validity and reliability of the FFQ has been examined in a number of studies [25-27]. A recent study found that all of the nutrient estimates from the FFQ were within ± 20% of the estimates produced from the mean of the 3-day weighed food records in adults [27]. Another study found that the FFQ could be used to rank subjects according to their likely plasma n-3, DHA, and n-6 fatty acid intake and the ratio of n-6: n-3 fatty acids.

Yamax SW700 pedometers (Yamax Corporation, Kumamoto City, Japan) were used to provide an objective measure of physical activity. At baseline assessments, participants were instructed on how to attach the pedometers (at the waist on the right hand side) and asked to remove the pedometers only when sleeping, when the pedometer might get wet (e.g. swimming, showering) or during contact sports. At the end of the day participants were instructed to record their steps and reset their pedometers to zero. If the participants forgot to wear their pedometers or removed their pedometers for more than 2 hours during the day, participants were instructed to leave their log sheet blank. Once they had completed seven days of monitoring, participants were instructed to place the pedometer and record sheet in the prepaid envelope provided and return to the research team. Physical activity variability was explored using intraclass correlation coefficients (ICCs).

### Analysis

The data were analyzed with SPSS^® ^version 16.0 (SPSS Inc., Chicago, IL). Last observation carried forward was used for missing data (i.e. baseline or 3-month scores carried forward). Both intention-to-treat and planned per-protocol mediation analyses were conducted. Of the 34 participants assigned to the Internet group, 14 (41.2%) complied well with treatment, defined as seven weeks of submission of daily eating and exercise diaries (*n *> 50) and weekly check-ins (*n *> 12) over the 3-month period. In the per-protocol mediation analysis, the 'compliers' were compared to the control group. Measures of change in weight from baseline to 6-month posttest were computed by regressing the posttest values onto their baseline values to create residualized weight change indices [28,29]. Using residualized change scores addresses the issue of regression to the mean [30] and accounts for the possibility that men classified as obese at baseline have more weight to lose than overweight participants. The same process was used for hypothesized mediators (e.g. physical activity, fat intake).

A product-of-coefficients test was used to assess mediation in the SHED-IT intervention because it has good statistical power in small samples [31-33] and can be used to establish mediation even in the absence of a statistically significant intervention effect [34]. Asymmetric confidence intervals were used to test the significance of the product of coefficients tests as they are more accurate than normal confidence limits [35]. Single mediator models were tested using ordinary least squares regression. The mediators assessed in this study were based on the SHED-IT weight loss tips and included the following: physical activity, portion size, total energy intake, alcohol intake and fat intake. To determine whether each variable mediated weight loss, the following regression models were calculated. First, the 'total effect' of the intervention on weight, controlling for baseline was examined (τ). Second, the effect of the intervention on hypothesized mediators was assessed (α). In the third step, treatment and hypothesized mediators were both entered into a regression model predicting weight (β). The mediated effect was calculated by multiplying α and β. In the final step, asymmetric confidence intervals were used to test the significance of the product of coefficients (αβ) using Mackinnon et al.'s PRODCLIN program [36]. If zero is outside the confidence interval, then the mediated effect is statistically significant [34].

## Results

### Baseline data

Measurements were obtained for 83% of the sample at 6-months (n = 54). There was no difference in follow-up rates between the Internet and control groups at 6-months (χ^2 ^= .03, *df *= 1, *P *= .87). There were no significant differences in baseline characteristics between those lost to follow-up and those retained at 6-months for age, weight or any of the secondary outcomes (*p *> .05). Table 1 presents baseline characteristics of the sample highlighting no difference by group. The age range was 19 to 59 years and comprised 43% students, 41.5% non-academic staff and 15.4% academic staff. Of the study participants, 62 (95%) completed a usable FFQ at baseline, 54 (83%) at 3-months and 53 (82%) at 6-months. Fifty nine (91%) provided step counts at baseline, 50 (77%) at 3-months and 39 (60%) at 6-months. Last observation carried forward was imputed for missing values. Baseline and posttest scores are reported in Table 2. The mean weight was 99.1 kg (12.8) and 52.3% of the sample were obese (BMI>30). The intraclass correlation coefficient (ICC) (95% confidence intervals) for mean steps/day was 0.82 (0.74 to 0.88) for seven days.

**Table 1:** Baseline characteristics of men randomized to the control and Internet groups

	Control (n = 31)		Intervention (n = 34)		Total (N = 65)	
	
Characteristics	Mean	(SD)	Mean	(SD)	Mean	(SD)
Age (years)	34.0	11.6	37.5	10.4	35.9	11.1
*Occupation, n (%)*						
Student	14	45.1	14	41.2	28	43.0
Non-academic staff	13	41.9	14	41.2	27	41.5
Academic staff	4	12.9	6	17.6	10	15.4
*SES*^*a *^, *n (%)*						
1-2 (lowest)	0	0.0	1	4.2	1	1.9
3-4	5	17.9	7	29.2	12	23.1
5-6	9	32.1	3	12.5	12	23.1
7-8	11	39.3	11	45.8	22	42.3
9-10 (highest)	3	10.7	2	8.3	5	9.6
Weight (kg)	99.2	13.7	99.1	12.2	99.1	12.8
Height (m)	1.8	0.1	1.8	0.1	1.8	0.1
BMI (kg/m^2)^	30.5	3.0	30.6	2.7	30.6	2.8
*BMI Category*						
Overweight, n (%)	15	48.4	16	47.1	31	47.7
Obese, n (%)	16	51.6	18	52.9	34	52.3

Abbreviations: BMI = Body Mass Index; SES = socioeconomic status

a SES by population decile for SEIFA Index of Relative Socio-economic Advantage and Disadvantage

Overweight classified as BMI ≥ 25 kg/m^2^; Obese classified as BMI ≥ 30 kg/m^2^

**Table 2:** Specific values for pretest and posttest scores in the SHED-IT study

	Baseline		6-month follow-up	
	
Variable	CON (n = 31)	INT (n = 34)	CON (n = 31)	INT (n = 34)
Weight (kg)	99.16 (13.80)	99.10 (12.22)	95.14 (13.80)	93.13 (15.16)
Physical activity (steps/day)	8102 (2615)	8869 (2573)	8933 (3522)	9778 (2452)
Portion size (portion size factor)^*a*^	1.51 (0.42)	1.51 (0.39)	1.25 (0.37)	1.30 (0.39)
Total energy (kJ/day)	9271 (3206)	11027 (3550)	7791 (3258)	8115 (2667)
Alcohol (gm/day)	14.13 (15.00)	21.83 (21.07)	13.73 (15.99)	15.46 (14.01)
Total fat (gm/day)	100.84 (34.21)	112.35 (38.95)	81.20 (41.29)	77.98 (30.02)

Abbreviations: CON = Control, INT = Intervention, Means reported and standard deviations in brackets.

^*a *^Portion size photographs are used to calculated a single portion size (PSF) factor is to indicate whether on average a person eats median size serves (PSF = 1), more than the median (PSF > 1), or less (PSF < 1) and is used to scale the serve size for vegetables, meat and casseroles.

### Intention-to-treat mediation analyses

Both groups lost weight, but relative to the control group, the Internet intervention did not have a statistically significant 'total effect' on weight, τ = -.507, *p *= .716 (95% CI = -3.277 to 2.263). The relationships between treatment and hypothesized mediators are reported in Table 3. The effects of hypothesized mediators on weight are reported in Table 4. The intervention did not have a statistically significant effect on any of the hypothesized mediators and none of the mediators were significantly associated with weight. Consequently, none of the hypothesized mediators satisfied the criteria for mediation. Mediated effects and asymmetric confidence intervals are reported in Table 5.

**Table 3:** Effect of treatment condition on hypothesized mediators (α)

Variable	Intention-to-treat analysis		Per-protocol analysis	
	
	α **(SE)**	95% CI	α **(SE)**	95% CI
Physical activity (steps/day)	229 (570)	-912 to 1369	24 (730)	-1452 to 1501
Portion size (PSF)	.06 (.07)	-.077 to .188	.06 (.09)	-.13 to .24
Total energy (kJ/day)	-449 (668)	-1786 to 888	-649 (847)	-2361 to 1063
Alcohol (gm/day)	-3.05 (2.31)	-7.67 to 1.58	-.549 (3.26)	-7.13 to 6.03
Total fat (gm/day)	-8.80 (7.73)	-24.27 to 6.66	-11.31 (9.86)	-31.24 to 8.62

Abbreviations: α = estimate of unstandardised regression coefficient effect of intervention on hypothesized mediators controlling for baseline; SE = standard error; 95% CI = 95% confidence interval

**Table 4:** Effect of hypothesized mediators on weight controlling for treatment allocation (β)

Variable	Intention-to-treat analysis		Per-protocol analysis	
	
	β **(SE)**	95% CI	β **(SE)**	95% CI
Physical activity	.0003 (.0003)	-.0010 to .0003	.0006 (.0003)	-.0014 to .0002
Portion size	3.05 (2.63)	-2.21 to 8.31	2.91 (3.00)	-3.15 to 8.79
Total energy	.0003 (.0003)	-.0002 to .0009	.0001 (.0004)	-.0006 to .0009
Alcohol	.027 (.081)	-.135 to .189	.049 (.093)	-.140 to .238
Total fat	.052 (.023)	.006 to .099	.045 (.030)	-.016 to .106

Abbreviations: β = estimate of unstandardised regression coefficient of mediator with treatment condition included in the model; SE = standard error; 95% CI = 95% confidence interval.

**Table 5:** Mediated effect and asymmetric confidence intervals (αβ)

Variable	Intention-to-treat analysis				Per-protocol analysis			
	
	αβ	ProdLower	ProdUpper	95% CI	αβ	ProdLower	ProdUpper	95% CI
Physical activity	.07	-1.87	2.40	-.28 to .51	.01	-2.09	2.12	-.90 to .94
Portion size	.18	-1.60	2.47	-.24 to .84	.17	-1.70	2.48	-.37 to .96
Total energy	-.13	-2.47	1.69	-.73 to .27	-.06	-2.35	1.99	-.71 to .48
Alcohol	-.08	-2.33	1.94	-.68 to .41	-.03	-2.29	2.07	-.41 to .32
Total fat	-.46	-2.33	1.64	-1.51 to .28	-.51	-2.44	1.51	-1.87 to .33

Abbreviations: αβ = product of coefficients estimate; ProdUpper and ProdLower represent the upper and lower critical values obtained from the PRODCLIN program; 95% CI = 95% asymmetric confidence intervals for the mediated effect obtained from the PRODCLIN program.

### Per-protocol mediation analysis

In the per-protocol analysis, the Internet intervention had a statistically significant 'total effect' on weight, τ = -4.487, *p *< .05 (95% CI = -8.208 to -.765). Relationships between mediators, treatment and outcome variables are reported in Tables 3 and 4. Similar to the intention-to-treat analysis results, the intervention did not have a statistically significant effect on any of the hypothesized mediators and none of the mediators satisfied the criteria for mediation (Table 5).

## Discussion

Few studies have examined potential mediators of weight loss in obesity treatment interventions and this is the first study to explore behavioral mediators in an intervention for overweight men. A product-of-coefficients test was used to assess mediation in the current study because it can be used to establish mediation in small samples, even in the absence of a statistically significant intervention effect [31-34]. In the current study, none of the hypothesized behavioral variables satisfied the criteria for mediation. While this finding is discouraging, it is important for obesity treatment and prevention trials to perform and report mediation analyses even in the presence of null findings [7]. Mediation analyses can help identify sources of methodological or substantive problems [7] and thus improve the design of future interventions.

Estimates of total energy intake decreased by almost 3,000 kJ over the study period, but did not satisfy the criteria for mediation. This suggests that reductions in intake may have contributed to weight loss or that energy intake was confounded by misreporting. In a previous on-line intervention for overweight adults [12], changes in dietary intake were related to weight loss in the behavior therapy and education groups at 3- and 6-months, respectively. In the current study, men in the intervention group reduced their fat in-take, but the reductions did not satisfy the criteria for mediation. Findings from previous weight loss interventions have been mixed. A recent Internet-based intervention had a modest effect on weight loss (-1.3 kg) and no effect on fat intake in a sample of overweight adults [37]. Another study involving overweight African American adolescent girls and their parents [16] found that reductions in fat intake partially mediated changes in BMI among parents in the study. Future versions of the SHED-IT intervention may benefit from revised behavioral messages that include reducing fat intake. Furthermore, tailored feedback focusing on strategies to reduce fat intake may improve weight loss among overweight men.

Although the SHED-IT intervention was a minimal contact program and did not include a delivered physical activity component, increasing lifestyle physical activity was one of the key SHED-IT aims and the intervention included two weight loss tips focusing on the promotion of physical activity (i.e. every step counts and tilt the balance with physical activity). However, changes in physical activity were not related to weight loss in both intention-to-treat and per-protocol analyses, as participants in both groups increased their physical activity over the study period [21]. Previous Internet-based interventions have found that changes in physical activity were related to weight loss among overweight women [11] and overweight adults [12]. The use of unsealed pedometers in the assessment of physical activity may have confounded the results and provided inaccurate and or inflated step counts, particularly at baseline. Pedometers have been successfully used in a number of interventions to increase physical activity using self-monitoring strategies with adults [38], children and adolescents [39]. The basic premise of self-monitoring with pedometers is that the daily step counts act as a reminder of the amount of activity completed by the individual and this in turn, encourages the individual to increase their physical activity.

Participants in the intervention reduced their alcohol intake over the study period, but these changes were not related to weight loss in the intention-to-treat or per-protocol analyses. An important aspect of mediation analysis is that it enables researchers to identify if changes in an intermediary variable contributed to changes in the dependent variable of interest. Studies that fail to use mediation analyses may incorrectly attribute weight loss to unrelated or less important behaviors.

In the current study we did not directly assess the participants' self-monitoring of physical activity and eating at baseline and posttest. However, in the per-protocol analysis, participants who adhered to the on-line tracking component of the intervention were more successful in losing weight compared to non-compliers. Self-management strategies such as goal setting and self-monitoring are important for successful weight loss and maintenance of weight loss [40], so it is not surprising that interventions which facilitate higher rates of self-monitoring result in improved behavior change outcomes [41]. For example, in a study involving overweight adults, Tate and colleagues [42] found that login rates were significantly correlated with weight change in their e-counseling intervention (*r *= -.47, *p *= .003) and their basic Internet intervention (*r *= -.61, *p *< .001). The identification of techniques to enhance the attractiveness of self-monitoring strategies in on-line weight loss programs should be a research priority.

This study has some limitations that should be noted. First, the study did not include a true control and both groups significantly reduced their weight over the study period. Consequently, the results of our mediation analysis were confounded by the significant reductions in weight experienced by the control group. While the control group received only one information session and booklet focused on weight loss strategies, there were no statistically significant differences in weight loss between intervention and control groups after 6-months. Second, the study lacked statistical power to detect small mediation effects in the study sample and hence a type 2 error may have occurred. Although traditional methods to evaluate mediation, such as the Baron-Kenny method [43] have low statistical power and require large samples, simulation studies have demonstrated that various other tests of mediation can establish statistically significant mediation effects in smaller samples [32,44]. Third, the current study found only small changes in portion size and these were identified in both the intervention and control groups. There was only a small range of portion sizes reported and future studies may benefit from the inclusion of a more detailed measure of portion size. Fourth, it is possible that some diffusion of treatment may have occurred, as both control and intervention participants were staff and students from the University of Newcastle. Finally, the study did not assess all of the potential weight loss mediators, including 'surf the urge', 'plan your meals' and 'reduce your junk food snacks'. It is possible that the SHED-IT intervention impacted upon these behaviors which in turn contributed to the participants' weight loss.

## Conclusion

Although previous studies have examined psychosocial mediators of weight loss in interventions [16], this is the first study to assess behavioral mediators in an intervention for overweight men. Like many obesity prevention and treatment programs, the SHED-IT intervention was based on key behavioral messages related to weight loss and maintenance. The identification of behavioral mediators of weight loss can be used to develop an evidence base of effective weight loss strategies in specific target groups. Further study of psychosocial and behavioral mediators within weight loss studies may help to refine effective intervention components to enhance weight loss outcomes.

## Competing interests

The authors declare that they have no competing interests.

## Authors' contributions

PJM, DRL, RC and CEC obtained funding for the research. All authors contributed to developing the protocols and reviewing, editing, and approving the final version of the paper. PJM and JMW were responsible for drafting the written materials. The trial was implemented by PJM and CEC, JMW, DRL and RC provided advice and guidance on the strategies and conduct of the RCT. DRL and RC were responsible for data collection. DRL conducted the analysis and drafted the first version of the manuscript. PJM is the guarantor and accepts full responsibility for the conduct of the study and the integrity of the data. DRL is responsible for the accuracy of the data analysis.
